# Remaining Useful Life Estimation for Engineered Systems Operating under Uncertainty with Causal GraphNets

**DOI:** 10.3390/s21196325

**Published:** 2021-09-22

**Authors:** Charilaos Mylonas, Eleni Chatzi

**Affiliations:** Department of Civil, Environmental and Geomatic Engineering, Stefano-Franscini-Platz 5, 8093 Zürich, Switzerland; chatzi@ibk.baug.ethz.ch

**Keywords:** ball bearings, condition monitoring, forecast uncertainty, Graph Neural Networks (GNNs), Recurrent Neural Networks (RNNs), non-uniform sampling, Remaining Useful Life (RUL)

## Abstract

In this work, a novel approach, termed GNN-tCNN, is presented for the construction and training of Remaining Useful Life (RUL) models. The method exploits Graph Neural Networks (GNNs) and deals with the problem of efficiently learning from time series with non-equidistant observations, which may span multiple temporal scales. The efficacy of the method is demonstrated on a simulated stochastic degradation dataset and on a real-world accelerated life testing dataset for ball-bearings. The proposed method learns a model that describes the evolution of the system implicitly rather than at the raw observation level and is based on message-passing neural networks, which encode the irregularly sampled causal structure. The proposed approach is compared to a recurrent network with a temporal convolutional feature extractor head (LSTM-tCNN), which forms a viable alternative for the problem considered. Finally, by taking advantage of recent advances in the computation of reparametrization gradients for learning probability distributions, a simple, yet efficient, technique is employed for representing prediction uncertainty as a gamma distribution over RUL predictions.

## 1. Introduction

Predictive tasks relying on time series data are encountered in diverse technological and scientific fields. A prominent application in this respect lies in the assessment of the remaining useful life of structural components and industrial assets, such as bearings [1,2,3]. For a number of these predictive tasks, observations are available only in non-equidistant and sparse intervals. In these problems, additional assumptions on the evolution of these time series are necessary for facilitating prognostic tasks [4]. Physics-based models that are able to simulate the evolution of the underlying systems can offer a solution to the problem of missing and non-equidistant timeseries data, by completing knowledge through simulation. However, these are typically either unavailable, of lower precision, or associated with prohibitively expensive numerical computations and/or modeling effort. On the other hand, large volumes of measurement data that correlate with Quantities of Interest (QoIs) in a non-trivial manner are often readily available. Moreover, when the evolution of the system at hand is non-deterministic, even if a perfect knowledge of the instantaneous system state (condition) is somehow achieved, a deterministic estimate of the long-term evolution of the system is still non-trivial due to the accumulating uncertainty for future predictions. Therefore, in the case of stochastically evolving systems, it is important to account for the uncertainty that is inherently present. The problem of Remaining Useful Life (RUL) prediction, which is tackled in this work, falls in the class of stochastically evolving systems, where data at hand are often missing or non-equidistant.

In many real-world applications, as in the case-studies examined herein, a model of degradation and final failure is not available or not reliable enough. At this point, a clear distinction should be made between the (long-term) model of degradation of a component and the (short-term) model of the time series of the dynamic response of the component. We consider settings where none of these models are available but where raw measurements of the time-series response data over shorter intervals are available, along with logged failure times. This work proposes fully data-driven methods, where a model is to be implicitly learned directly from field or experimental observations. Note that the physics of mechanical systems, such as ball bearings, are relatively well understood [5]. However, the uncertainty which characterizes the various parameters that are involved in a detailed physics-based analysis, makes the rigorous assessment of the stochastic RUL problem harder. Such uncertainties are manufacturing imperfections, the effects of environmental conditions on frictional properties, material and manufacturing imperfections, as well as the noisy, often lacking logging of loading conditions. In the same context, it is expected that features related to the damage of the components evolve stochastically and that the progression of damage state has an indirect effect on the observed raw time series.

The method proposed herein is inspired by the recent advances in GraphNets (GNs) [6], a framework for unification of certain classes of Graph Neural Networks (GNNs), and the flexibility these allow for in terms of defining relational inductive biases. Relational inductive biases are implemented by arranging data in an attributed graph. The most relevant architecture related to GNs are Message-Passing Neural Networks [7] but other proposed variants such as Non-local Neural Networks [8] can be cast into the GN framework. An inductive bias (or learning bias) is any belief or assumption that, when incorporated in the training procedure, can facilitate a machine learning algorithm to learn with fewer data or better generalize in unseen settings. In practice, for the problem of RUL estimation, due to interruptions in transmission or storage limitations, monitored time series (e.g., accelerations or strain measurements) contain gaps [9]. The non-regular sampling of the time series data is routinely treated as a missing data problem—a task most commonly referred to as time series imputation [10,11,12]. This often requires to impose an explicit evolution model that reproduces the raw time series itself in regular intervals, so that algorithms designed to work with data observed in regular intervals can be used. This approach biases the subsequent treatment of the data with predictive algorithms. The present work, in addition to providing a solution to long time series, proposes a radically different yet more natural approach to the problem of irregular observations for RUL predictive models. Instead of completing the missing data and subsequently employing a time series technique that operates on equidistant data, a model is directly learned from the available non-regularly spaced data. Instead of imposing an explicit model of the time series, the temporal ordering of the observations is incorporated in the learning algorithm as an inductive bias.

Incorporation of inductive biases is useful in constructing machine learning models that need to be trained on relatively small datasets and for building smaller and more computationally efficient models [6]. Recurrent Neural Networks (RNNs) impose a chain-structure of dependence, which constrains them to sequential computations. When considering long time-series, this becomes a significant computational disadvantage both in training and evaluation of RNNs. Furthermore, owing to their chain-structured sequential computation, it is difficult for RNNs to transfer information from distant past states to current or future estimates. Namely, for propagating information *N* steps ahead, *N* steps of sequential computation need to be performed. A highly effective remedy for the sequential computation of RNNs was delivered via the introduction of attention mechanisms [13], which led to great advances in speech transcription [14] and neural machine translation [15]. Attention mechanisms allow the model to attend to a large finite window of observations, therefore relieving the burden of propagating information forward sequentially. Moreover, RNNs rely on the feeding of discrete-time and equidistant data. Recent approaches to sequence modeling for non-equidistant data such as NeuralODEs [16] and Legendre Memory Units [17] offer solutions to the problem of irregular data, but do not facilitate the easier propagation of information from arbitrary past steps, since they retain the chain structure of RNNs. In contrast to RNNs, the architecture proposed in this work, termed Graph Network with Temporal CNN feature extractors GNN-tCNN, does not assume a chain graph for processing the past time-steps, but instead adopts a more general causal graph.

It is noted that the non-sequential processing of ordered data has further prompted several important advancements in the field of Natural Language Processing (NLP), which led to the complete removal of RNN components from NLP architectures and their replacement with the transformer architecture [18]. In addition to their success in NLP domain, transformers have been extended to general multivariate time series representation learning [19]. Similarly to the transformer architecture, the proposed GNN-based architecture operates in a parallelized manner with a constant (and adjustable) number of sequential computational steps, as will be detailed in Section 3. Owing to the GNN-based processing, the proposed model naturally allows for arbitrarily spaced data which is a feature that separates this work from other transformer-based RUL architectures.

### Machine Learning for Time Series and RUL

Classical machine learning techniques for time series datasets consist of separate feature extraction and selection and predictive model selection pipelines. The most widely used feature extraction techniques, naturally fitting to time series models, are (1) discrete fourier transforms, due to the intuitive decomposition of the signal to coefficients (2), wavelet transforms [20,21], and (3) dynamic time-warping, when the main source of variation among signals is due to some temporal distortion (i.e., non-stationarity), such as different heart-rates in EEG classification [22] or different rotational speeds in machinery [23]. In several applications of machine learning for predictive time series models, simple statistical moments of the signals are used, such as kurtosis and standard deviation of time series segments [24]. For some special applications, expert-guided feature extraction techniques have been proposed to facilitate downstream tasks. One successful representative example of this class of models, within the context of time series analysis, are Mel-frequency cepstral features (MFCCs) [25] which are special to human auditory processing tasks (e.g., speech and music processing). In most typical applications found in the context of Structural Health Monitoring (SHM) the classical machine learning workflow is followed, where a large set of features are pre-computed and, in a second stage, features are selected by inspecting the generalization performance of the model (for instance with cross-validation). When physical intuition is not easy to draw from for the problem at hand, features are extracted by unsupervised learning techniques [26], such as autoencoders, or special negative-sampling based losses, such as time-contrastive learning [27]. Combinations of unsupervised learning techniques (such as autoencoders and deep Boltzmann machines) and hand-crafted pre-processing with discrete cosine transform are also used [28]. This classical ML workflow has been followed in several works related to condition monitoring and RUL estimation for bearings ([29,30,31]).

A number of recent works on RUL estimation for bearings adopt deep learning for the RUL prediction problem from time series data [32,33,34]. In [35], two CNN-based predictors are trained. One classifier predicts the point in time where a sudden increase in the amplitude of accelerations occurs, and which typically lies close to failure, and subsequently a second classifier predicts the time-to-failure after that point. The same approach is followed in [36], where Random Forests and XGBoost are used as predictive models. In [37], a recurrent convolutional network is adopted [38] and Monte-Carlo Dropout [39] is used as a simple and effective way of representing the uncertainty in the predictions. In [40], instead of recurrent connections, as applied in [38], attention layers are used to enhance the performance of CNNs. Chen et al. [41] propose an RNN-based architecture comprising an encoder–decoder structure with attention mechanism. The network is trained on band-passed energy values inferred from the frequency spectrum of vertical and horizontal vibration signals. Similarly, Hinchi and Tkiouat [1] propose an RUL estimation framework relying on convolutional and Long Short-Term Memory (LSTM) recurrent units. Li et al. [42] apply multi-scale feature extraction on time-frequency information collected from a short-time Fourier transform, while Ren et al. [43] employ collaborative prediction on both time and frequency domain features. All aforementioned approaches are not appropriate for non-equidistant data, as there is no explicit representation of the time between the observations. In the present work, a uniform treatment of the different stages of degradation is proposed without attempting to classify these stages since these are not clearly defined and this approach could unfavorably bias the results. In summary, the contributions of this work are as follows:

- A learnable state evolution model is proposed in order to account for the evolution of the deterioration process without explicitly forcing an underlying deterioration law.
- The non-stationary nature of the deterioration phenomenon is taken on by a latent state-space, which better capture the underlying non-linearity effects and is able to operate on non-equidistant observations.
- The function relating observations of segments of the time series to the (implicit) state-space and the function describing the long-term evolution of the time series is learned in an end-to-end differentiable manner, allowing for mini-batched training with stochastic gradient descent which scales favorably when dealing with large datasets.

## 2. Materials and Methods

### 2.1. A Simulated Degradation Process Dataset

In order to verify the efficacy of the method for RUL prediction tasks over long time series, a synthetic non-stationary degradation process was firstly simulated.

The purpose of generating such a controlled dataset is to verify the efficacy of the approach on a case study where the ground truth is known. The underlying process governing the degradation is assumed to follow a non-stationary Markov process with Gamma distributed increments [44]. This is of course not an exclusive assumption; further models have been shown to be suited for the simulating the RUL problem, including nonlinear Wiener Process models [45]. The parameters of the Gamma distributions producing the increments, which correspond to the deterioration of condition, are assumed to depend on the previous steps, as required by the physics of the deterioration phenomenon. In physical terms, this simulates the path dependence of irreversible processes. The random process presented herein does not have a direct physical analog and is only designed to demonstrate the ingredients of the proposed algorithm and verify its performance. The process generating the latent space is defined as follows

$$\begin{array}{cc} \delta \eta_{t_{i}}^{\left(\alpha ,\beta \right)} & ∼Gamma\left(\alpha \left(t_{i},c\right),\beta \right),\;\alpha \left(t,c\right)=0.02+t^{c}\\ z_{t_{k}}^{\left(\alpha ,\beta \right)} & =\sum_{i=0}^{t_{k}}\delta \eta_{t_{i}}^{\left(\alpha ,\beta \right)},\;z_{t_{k}}^{\left(\alpha ,\beta \right)}<z_{f} \end{array}$$

where $\left\{t_{0},t_{1},\cdots t_{N}\right\}$ are consecutive, discrete time steps, $\eta_{t}^{\left(\alpha ,\beta \right)}$ is a random variable with a non-linear dependence on time, and *c* is a random variable, which is assumed different for each experiment in order to reflect variability. The parameters α>0 and β>0 are termed the concentration or shape and rate parameters of the Gamma distribution. The probability density function of a Gamma distribution is defined as $f\left(x;\alpha ,\beta \right)=\frac{\beta }{\Gamma (\alpha )}x^{\alpha -1}e^{-x\beta }$, where Γ(·) denotes the Gamma function. Failure occurs when the latent accumulating damage variable $z_{t_{k}}$ reaches a threshold value $z_{f}$, which is assumed identical for all experiments. It is assumed that the different experiments feature slightly different damage evolution paths, which in practice reflects the variability induced due to manufacturing imprecision or errors. This is simulated by sampling *c* from a Gaussian distribution. The non-linear dependence is realized through the shape parameter α(t,c) of the Gamma distribution which controls the size of the increments. It should be noted that the non-linear dependence on time is adopted in order to simulate the non-stationarity of the process, arising due to dependence of “α” on the accumulated $z_{t_{k}}$. The high-frequency instantaneous measurement of the signal is denoted as $x_{t_{k}}$. The observations of the process consist of 1000 samples that contain randomly placed spikes with an amplitude that non-linearly depends on $z_{t_{k}}$; a process denoted via G(·) in what follows:

(1) $$\begin{array}{cc} {\tilde{z}}_{t_{k}} & =z_{t_{k}}+\epsilon \\ x_{t_{k}} & =G({\tilde{z}}_{t_{k}})+\zeta \end{array}\;\begin{array}{cc} \epsilon & ∼N(0,{\sigma_{z}}^{2})\\ \zeta & ∼N(0,{\sigma_{x}}^{2}) \end{array}$$

Gaussian noise is added both to the raw signal observation $x_{t_{k}}$, as well as directly onto the latent variable $z_{t_{k}}$. ζ reflects the observation noise which is normally distributed with a zero mean and variance ${\sigma_{z}}^{2}$. The observation noise reflects the error that may be present due to measurement imprecission. ϵ, also follows a Gaussian with variance $\sigma_{x}^{2}$ and is added to the instantaneous latent damage state $z_{t_{k}}$ in order to model the fact that $z_{t_{k}}$ may not be accurately determinable from $x_{t_{k}}$ even in the absence of ζ. Note that since $G\left(\cdot \right),\;R\to R^{1000}$ is a non-linear vector function the noise ϵ and ζ cannot be combined.

**Figure 1:**
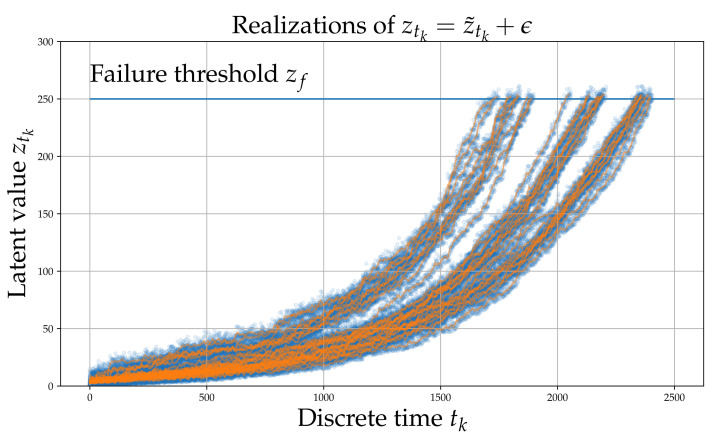
Simulated latent variable $z_{t_{k}}$. The blue points are $z_{t_{k}}$ whereas the orange line is ${\tilde{z}}_{t_{k}}$.

**Figure 2:**
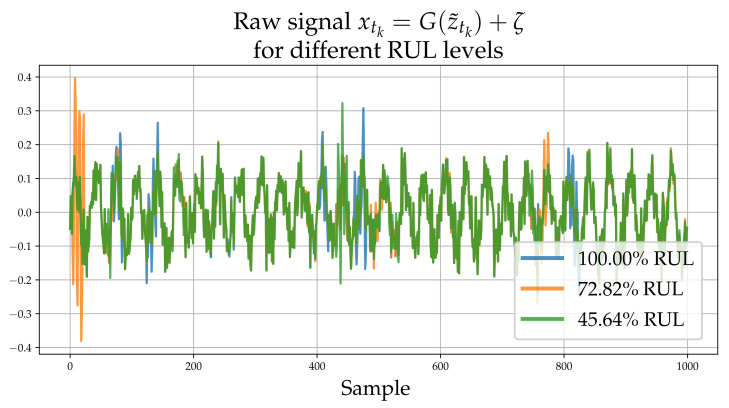
Raw high-frequency time series realizations $x_{t_{k}}$ corresponding to 100%, 72.82% and 45.64% for RUL. The process G(·) adds high frequency “spikes” of random magnitude which depend on ${\tilde{z}}_{t_{k}}$.

Each process underlying the observations of each experiment, evolves in the long-term in a similar yet sufficiently varied manner as shown in Figure 1. A set of $x_{t_{k}}$ signals (raw observations) are shown in Figure 2. The different colors correspond to different RULs. Although this process does not correspond directly to some actual physical problem, it is argued that it possesses all the necessary characteristics of a prototypical RUL problem and thus serves as a useful numerical case study that can aid verification.

### 2.2. An Experimental Dataset on Accelerated Fatigue of Ball Bearings

The PRONOSTIA dataset that is introduced in [46] consists of run-to-failure experiments for a total of 17 bearings, which have been loaded in 3 different rotational and lateral loading conditions which are summarized in Table 1. Only 2-axis acceleration measurements are used in the present work. Temperature measurements are further available but are not utilized in this work. Importantly, no artificial damage is introduced to the components for accelerating failure, thus rendering the accelerated testing scenario a better representation of real-world setting, where the failure mode is not known a priori.

In order to test generalization on un-seen experiments, a test-set containing whole experiments is used. A different train/test split is adopted from the standard cofiguration of the PRONOSTIA platform [46], as detailed in Table 2, in order to have a larger number of training experiments. Moreover, in the proposed split the distribution of total experiment durations between train and test set is more similar than in the original split and the three different loading conditions (A, B, and C) are represented in the train set. These properties are important for the employment of data-driven methods, since the statistics of predicted quantities and input quantities between train and test data should be similar.

Fatigue damage on ball bearings manifests as frictional wear of the bearings and/or the surrounding ring. Empirically, higher lateral loads $F_{i}$ and rotational speeds ${\dot{\phi }}_{i}$ are associated with faster wear. The ${\ddot{u}}_{x}$ and ${\ddot{u}}_{y}$ accelerometer data are available in 0.1 s segments, sampled at 25.6 kHz (2560 samples per segment). Temporal convolutional networks are used, in order to automatically learn features that are potentially useful for tracking degradation.

## 3. Model Architectures

### 3.1. GraphNets for Arbitrary Inductive Biases

**Figure 3:**
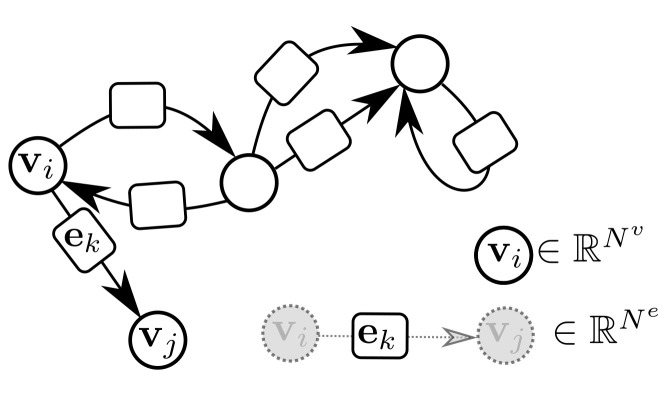
An attributed directed graph. Self-edges and multiple edges are allowed. Bi-directional edges are also allowed.

GraphNets (GNs) are a class of machine learning algorithms operating with (typically predefined) attributed graph data, which generalize several graph neural network architectures. An attributed graph, in essence, is a set of nodes (vertices) $V:\{v_{1},\cdots v_{k}\}$ and edges $E:\{\left(e_{1},r_{1},s_{1}\right)\cdots \left(e_{k},r_{k},s_{k}\right)\}$, with $e_{k}\in R^{N^{e}}$ and $v_{i}\in R^{N^{v}}$. Each edge is a triplet $\left(e_{j},r_{j},s_{j}\right)$ (or equivalently $\left(e_{j},v_{r_{j}},v_{s_{j}}\right)$) and contains a reference to a receiver node $v_{r_{j}}$, a sender node $v_{s_{j}}$ as well as a (vector) attribute $e_{j}$. Self-edges, i.e., when $r_{i}:=s_{i}$ are allowed. See Figure 3 for an example of an attributed graph.

In [6], a more general class of GraphNets is presented, where global variables which affect all nodes and edges are allowed. A GN with no global variables consists of a node-function $\phi^{v}$, an edge function $\phi^{e}$, and an edge aggregation function $\rho^{e\to v}$. The function $\rho^{e\to v}$ should be (1) invariant to the permutation of its inputs and (2) able to accept a variable number of inputs. In what follows this will be referred to as the edge aggregation function. Simple valid aggregation functions are Min(·), Max(·), Sum(·) and Mean(·). Inventing more general aggregation functions (for instance by combining them) and investigating how these affect the approximation properties of GNs currently forms an active research topic [47].

**Figure 4:**
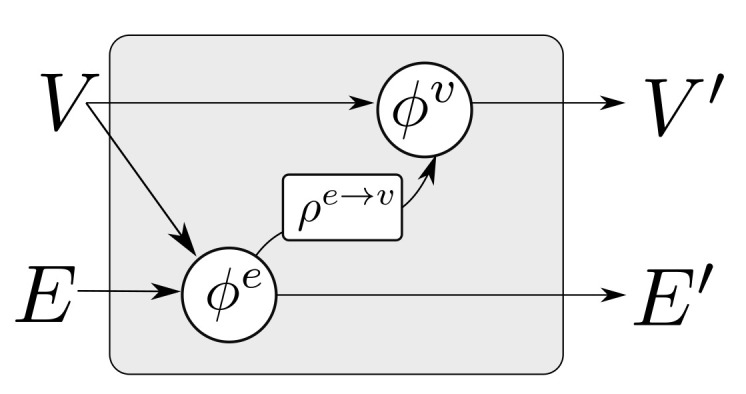
A single GN block with message passing. The block updates the set of edges $E\to E^{\prime }$ and nodes $V\to V^{\prime }$ according to Algorithm 1.

Ignoring global graph attributes, the GraphNet computation procedure is as detailed in Algorithm 1. First, the new edge states are evaluated using the sender and receiver vertex attributes ($v_{s_{i}}$ and $v_{r_{i}}$ correspondingly) and the previous edge state $e_{i}$ as arguments to the edge function $\phi^{e}$. The arguments of the edge function may contain any combination of the source and target node attributes and the edge attribute. Afterwards, the nodes of the graph are iterated and the incoming edges for each node are used to compute an aggregated incoming edge message ${\bar{e}}_{i}^{\prime }$. The aggregated edge message together with the node attributes are used to compute an updated node state. Typically, small Multi-Layer Perceptrons (MLPs) are used for the edge and node GraphNet functions $\phi^{e}$ and $\phi^{v}$. It is possible to compose GN blocks by using the output of a GN as the input to another GN block. Since a single GN block allows only first order neighbors to exchange messages, GN blocks are composed as

$$\begin{array}{c} GN_{K}\left(GN_{K-1}\left(\cdots \left(GN_{0}\left(G\right)\cdots \right)\right)\right)=\\ GN_{K}\circ GN_{K-1}\circ \cdots \circ GN_{0}\left(G\right) \end{array}$$

where “∘” denotes composition. The first GN block may cast the input graph data to a lower dimension so as to allow for more efficient computation. The first GN block may comprise edge functions that depend only on edge states $\phi^{e_{0}}\left(e\right)$ and correspondingly node functions that depend only on node states $\phi^{u_{0}}\left(v\right)$. This is referred to as a Graph Independent GN block and it is used as the type of layer for the first and the last GN block. The inner GN steps (i.e., $GN_{1}$ to $GN_{K-1}$) are full GN blocks, where message passing takes place. This general computational pattern is referred to as encode-process-decode [6]. The inner GN blocks have shared weights, yielding a lower memory footprint for the whole model, or can comprise different weights, which amount to different GN functions that need to be trained for each level. Sharing weights and repeatedly applying the same GN block helps propagate and combine information from more connected nodes in the graph. A message passing GN block which does not contain the global variable, as the ones used in this work, is shown in Figure 4.
**Algorithm 1:** GN block without global variables [6].**function**GraphNetwork (*E*, *V*)    **for** $k\in \{1\ldots N^{e}\}$ **do**        $e_{k}^{\prime }\leftarrow \phi^{e}\left(e_{k},v_{r_{k}},v_{s_{k}}\right)$                         ▹ 1. Compute updated edges    **end for**    **for** $i\in \{1\ldots N^{n}\}$ **do**        **let** $E_{i}^{\prime }={\left\{\left(e_{k}^{\prime },r_{k},s_{k}\right)\right\}}_{r_{k}=i,\;k=1:N^{e}}$        ${\bar{e}}_{i}^{\prime }\leftarrow \rho^{e\to v}\left(E_{i}^{\prime }\right)$                                    ▹ 2. Aggregate edges per node        $v_{i}^{\prime }\leftarrow \phi^{v}\left({\bar{e}}_{i}^{\prime },v_{i},\right)$                                    ▹ 3. Compute updated nodes    **end for**    **let** $V^{\prime }={\left\{v^{\prime }\right\}}_{i=1:N^{v}}$    **let** $E^{\prime }={\left\{\left(e_{k}^{\prime },r_{k},s_{k}\right)\right\}}_{k=1:N^{e}}$    **return** $\left(E^{\prime },V^{\prime }\right)$**end function**

In the present work, as is the case with RNNs [48] and causal CNNs [49], the causal structure of time series is exploited, which serves as a good inductive bias for the problem at hand, although without requiring that the data is processed as a chain-graph or that the data are equidistant. Instead, an arbitrary causal graph for the underlying state is built, together with functions to infer the quantity of interest which is the remaining useful life of a component given a set of non-consecutive short-term observations.

### 3.2. Incorporation of Temporal Causal Structure with GNs and Temporal CNNs (GNN-tCNN)

**Figure 5:**
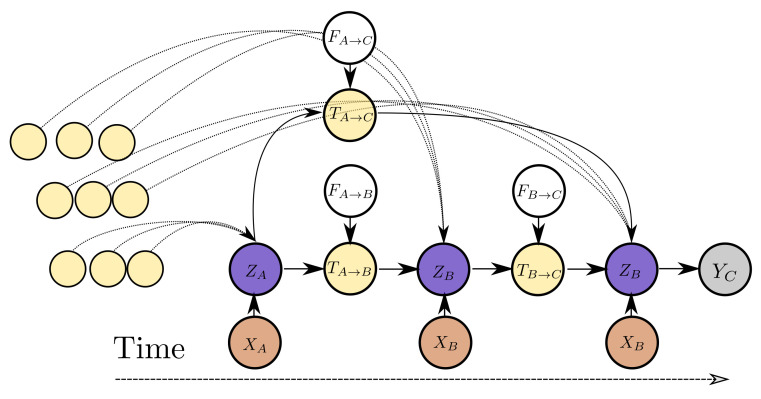
Dependency graph for the variables associated with the proposed model. $X_{A}$ represents the raw observed variable at time $t_{A}$. Variable $Z_{A}$ represents the (unobserved) state that can be translated to the quantity of interest $Y_{C}$ or a probabilistic estimate.

**Figure 6:**
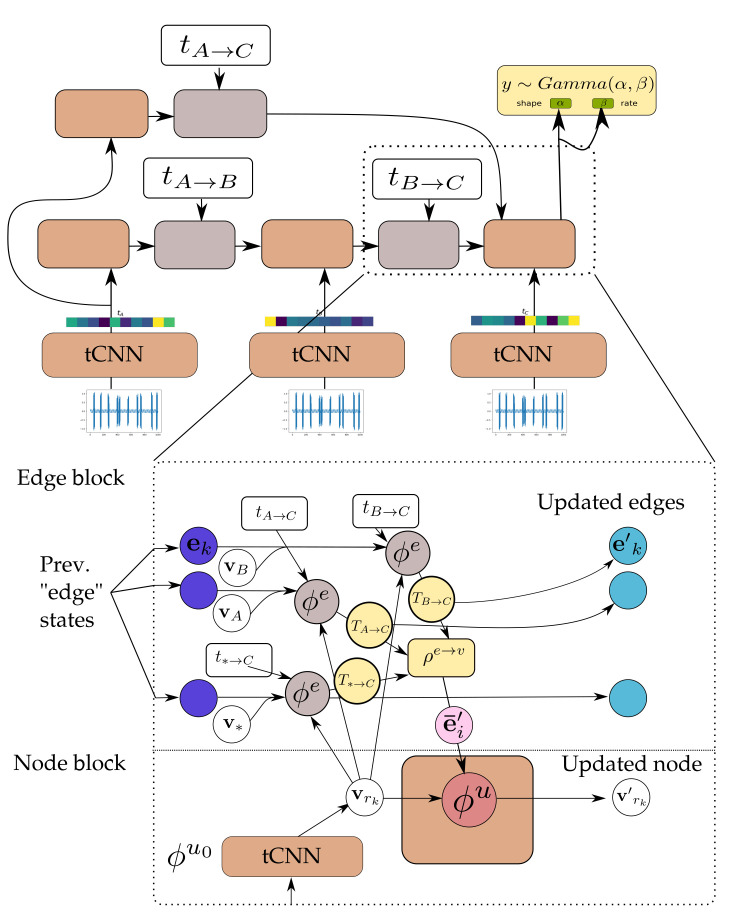
Detail of the **GNN-tCNN**. Nodes $v_{A},v_{B},v_{*}$ correspond to sender nodes for the respective edges. The updated edge states (light blue) are the same as the messages (yellow). See also Algorithm 1 and Figure 7.

The variable dependencies of the proposed model are depicted in Figure 5 for three observations. The computational architecture is depicted in detail in Figure 6. The variable $Z_{K}$ represents the current estimate for the latent state of the system. This corresponds to the node states *V*. The variable $T_{K\to L}$, which represents the propagated latent state from past observations, depends on the latent state $Z_{K}$, an exogenous input $F_{K\to L}$ that controls the propagation of state $Z_{K}$ to $Z_{L}$ and potentially other propagated latent state estimates from instants before $t_{L}$. The variable $T_{K\to L}$ corresponds to an updated edge state $E^{\prime }$ and the exogenous inputs $F_{L\to L}$ can be the edge state before the edge step *E*. The exogenous input $F_{K\to L}$ to the state propagation function can be as simple as the elapsed time between two time instants, i.e., $F_{K\to L}=t_{K\to L}=t_{L}-t_{K}$ or encode more complex inductive biases, such as the values representing different operating conditions during the interval between observations. An arbitrary number of past states can be propagated from past observations and aggregated in order to yield better estimates for a latent state $Z_{L}$. In addition to propagated latent states, instantaneous observations of raw data $X_{K}$ inform the latent state $Z_{K}$. For instance, in Figure 5, $Z_{C}$ depends on $T_{B\to C}$ but at the same time on $T_{A\to C}$ and potentially more propagated states from past observations (other yellow nodes in the graph) and at the same time to an instantaneous observation $X_{C}$. Each inferred latent state $Z_{i}$ can be transformed to a distribution for the quantity of interest $Y_{i}$. The value of the propagated state variable from state *s* to state *d*, $T_{s\to t}$, depends jointly on the edge attributes and on the latent state of the source node. In a conventional RNN model, $F_{K\to L}$ corresponds to an exogenous input for the RNN cell. In contrast to an RNN model, in this work the dependence of the estimate of each state depends on multiple states by introducing a propagated state that is modulated by the exogenous input. In this manner, an arbitrary and variable number of past states can be used directly for refining the estimate of the current latent state, instead of the estimate summarized in the latent cell state of the RNN. In the proposed model, the parameters of the functions relating the variables of the model are learned directly from the data and essentially define the inductive biases following naturally from the temporal ordering of the observations. This approach allows for uniform treatment of all observations from the past and allows for the consideration of an arbitrary number of such observations to yield an estimate of current latent state.

**Figure 7:**
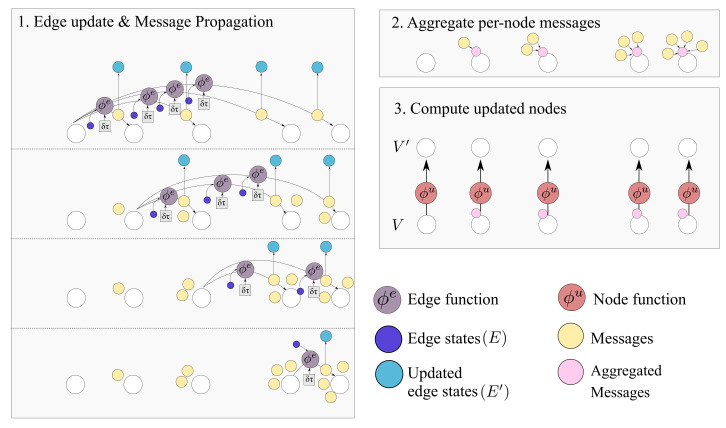
The Causal GN computation step. As detailed in Algorithm 1 the procedure can be summarized in edge update, aggregation and node update steps. The output graph contains $E^{\prime }$ and $V^{\prime }$. Several steps of this computation can be applied.

The connections from all observable past states and the ultimate one, where prediction (read-out) is performed, are implemented as a node-to-edge transformation and subsequent aggregations. Aggregation corresponds to the edge-aggregation function $\rho^{e\to u}\left(\cdot \right)$ of the GraphNet. In this manner, it is possible to propagate information from all distant past states on a single computation step. As mentioned also in the introduction, this is one of the computational advantages of the transformer architecture [18], which is related to GNs. In contrast to using a causal transformer architecture, the causal GNN approach proposed herein allows for parametrizing the edges between different states. This key difference is what allows the proposed model to work on arbitrarily spaced data. The different steps of the causal GN computation and how they relate to the general GN, are further detailed in Figure 7.

As is the case when using transformer layers, the computational burden increases quadratically with the context window. Therefore, the computation of all available past states would be inefficient. To remedy this, it is possible to randomly sample past observations in order to perform predictions for the current step. Similarly, during training, it is possible to yield unbiased estimates of gradients for the propagation and feature extraction model by randomly sampling the past states. It was found that for the presented use-cases this was an effective strategy for training.

In GN terms, the “encode” GraphNet block ($GN_{enc}:\left\{\phi^{u_{0}},\phi^{e_{0}}\right\}$) is a graph-independent block consisting of the node function $\phi^{u_{0}}$ and edge function $\phi^{e_{0}}$. The node function is a temporal convolutional neural network (temporal CNNs), with architecture detailed in Table 3.

The edge update function is a feed-forward neural network. The input of the edge function is the temporal difference between observations. Both networks cast their inputs to vectors of the same size. The $GN_{core}:\left\{\phi^{u_{c}},\phi^{e_{c}},\rho^{e\to u}\right\}$ network consists of small feed-forward neural networks for the node MLP $\phi^{u_{c}}$ and the edge MLP $\phi^{e_{c}}$. The input of the edge MLP is the sender and receiver state and the previous edge state. The MLP is implemented with a residual connection to allow for better propagation of gradients through multiple steps [50].

$$e_{i}^{\prime }\leftarrow \phi^{e_{c}}\left(e_{i},u_{s_{i}},u_{r_{i}}\right)={\bar{\phi }}^{e_{c}}\left(e_{i},u_{s_{i}},u_{r_{i}}\right)+e_{i}$$

In this work, the Mean(·) aggregation function was chosen, which does not depend strongly on the in-degree of the state nodes $Z_{i}$ (i.e., number of incoming messages) which corresponds to step 2 in Algorithm 1. The node MLP of the core network is also implemented as a residual MLP.

$$u_{i}^{\prime }\leftarrow \phi^{u_{c}}\left(u_{i},{\bar{e}}_{i}\right)={\bar{\phi }}^{u_{c}}\left(u_{i},{\bar{e}}_{i}\right)+u_{i}$$

The $GN_{core}$ network is applied multiple times to the output of $GN_{enc}$. This ammounts to the shared weights variant of GNs, which allows for propagation of information from multiple steps while costing a small memory footprint. After the last $GN_{core}$ step is applied, a final graph-independent layer is employed. At this point, only the final state of the last node is needed for further computation, i.e., the state corresponding to the last observation. The state of the last node is passed through two MLPs that terminate with Softplus activation functions

(2) Softplus(x)=log(exp(x)+1).

The Softplus activation is needed for forcing the outputs to be positive, since they are used as parameters for a Gamma distribution which in turn is used to represent the RUL estimates. The GraphNet computation procedure detailed above is denoted as

(3) $$g_{out}=GN_{tot}\left(g\right)=GN_{dec}\circ GN_{core}^{\left(N_{c}\right)}\circ GN_{enc}\left(g_{in}\right)$$

where $GN_{core}^{\left(N_{c}\right)}$ denotes $N_{c}$ compositions of the $GN_{core}$ GraphNet and “$g_{in},\;g_{out}$” are the input and output graphs. The vertex attribute of the final node as mentioned before is in turn used as the rate ($\alpha (GN_{tot}\left(g_{in}\right))$) and concentration ($\beta (GN_{tot}\left(g_{in}\right))$) parameters of a Gamma(α,β) distribution. For ease of notation, the parameters (weights) of all the functions involved are denoted by “θ” and the functions that return the rate and concentration are denoted as $f_{\alpha ;\theta }$ and $f_{\beta ;\theta }$ correspondingly to denote explicitly their dependence on “θ”. The Gamma distribution was chosen for the output values since they correspond to remaining time and they are necessarily positive. The GN described above is trained so as to directly maximize the expected likelihood of the remaining useful life estimates. For numerical reasons, equivalently, the negative log-likelihood (**NLL**) is maximized. The optimization problem reads,

$$\begin{array}{cc} \; & \underset{\theta }{arg max}E_{\left(P,S\right)}\left[p\left(y|g\right)\right]\propto \underset{\theta }{arg max}\prod_{i=1}^{N^{s,p}}p\left(y_{i}|g_{i}\right)\equiv \\ \; & =\underset{\theta }{arg min}\sum_{i=1}^{N^{s,p}}\left(-\log p(y_{i}|f_{\alpha ;\theta }\left(g_{i}\right),f_{\beta ;\theta }\left(g_{i}\right))\right) \end{array}$$

where g corresponds to the sets of input causal graphs, and y corresponds to the estimate of RUL for the last observation of each graph. The input graphs in our case consist of nodes, which correspond to observations and edges with time-difference as their features. Correspondingly $g_{i}$ and $y_{i}$ are single samples from the aforementioned set of causal graphs and remaining useful life estimates and $N^{s,p}$ denotes the number of sampled causal graphs from experiment *p* that are used for computing the loss (i.e., the batch size). The expectation symbol is approximated by an expectation over the set of available training experiments denoted as P and the random causal graphs created for training S. The gradients of Equation (5) are computable through implicit re-parametrization gradients [51]. This technique allows for low-variance estimates for the gradient of the NLL loss with respect to the parameters of the distribution, which in turn allows for a complete end-to-end differentiable training procedure for the proposed architecture.

### 3.3. Recurrent Neural Network with Temporal CNN Feature Extractors (LSTM-tCNN)

**Figure 8:**
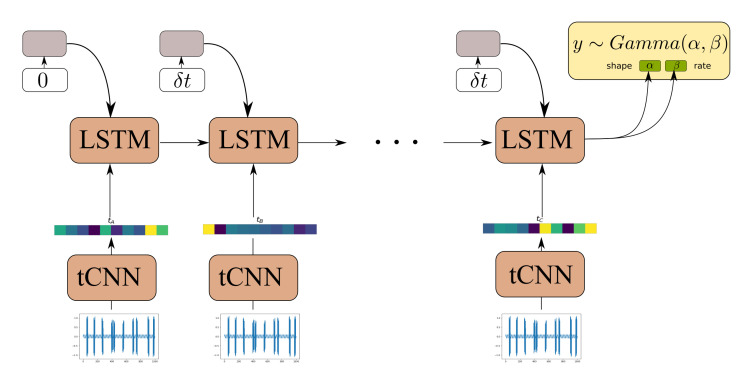
Model architecture of LSTM-tCNN.

The Causal GNN component of the architecture detailed in Section 3.2 is used to satisfy the following desiderata: (1) to allow for computationally efficient and parallelized propagation of information from time-instants in the distant past with respect to the current time step and (2) to allow for learning a state-propagation function and hence dealing with arbitrarily spaced points in a consistent manner. In order to offer a comparison to a further viable alternative, we here put forth a comparison of our proposed algorithm against an RNN-based approach. Although gated RNNs, such as GRUs and LSTMs, rely on sequential computation between time steps, and therefore are less parallelizeable, they are known to efficiently handle long dependencies. Moreover, by appending the time difference between observations in the RNN input gate the RNN allows the RNN to learn how to condition the predictions for the propagated state not only on the previous state and the CNN feature extractor input, but also to the time-difference between different RNN steps [52]. One such model, using an LSTM cell, is depicted in Figure 8.

### 3.4. Simple MLP Employed on Time Series Features

For completeness, and in order to provide a further benchmark comparison against baseline naive implementation, a simple three-layer ReLU MLP (100-units ReLU, Dropout, 100-units ReLU, 1-unit ReLU) with dropout rate 0.2 is used, and trained using the Mean Absolute Percentage Error (MAPE) loss, and using the features derived for each segment of the time series, separately. The derived features are summarized in Table 4. The MAPE loss reads,

(4) $$MAPE=\frac{100}{N}\sum_{t}^{N}\frac{|{\hat{RUL}}_{t}-RUL_{t}|}{RUL_{t}}$$

where $RUL_{t}$ corresponds to the actual remaining useful life for time series segment at time *t* and ${\hat{RUL}}_{t}$ refers to the MLP prediction and *N* is the number of time instants we use for the computation.

## 4. Results

### 4.1. Preliminary Architecture Selection for GNN-tCNN

As typically carried out for the case of recurrent neural network models [48,49,53], a gated-tanh activation function was used for the edge update and node update core networks.

$$h\left(y\right)=sigmoid\left(W_{g}y\right)⊙tanh\left(W_{a}y\right)$$

In preliminary results on the real dataset, GNs using this activation strongly outperformed the ones using tanh, but showed similar performance to the ones using ReLU activation. Networks for the edge and node MLPs were tested with widths 30,50, and 100. The smaller networks tested (size 30) consistently outperformed networks with size 50 and exhibited performance on par with networks with size 100 for some cases. Thus, the 30-unit networks were selected for the presented results for both simulated and real data.

### 4.2. Simulated Dataset

**Figure 9:**
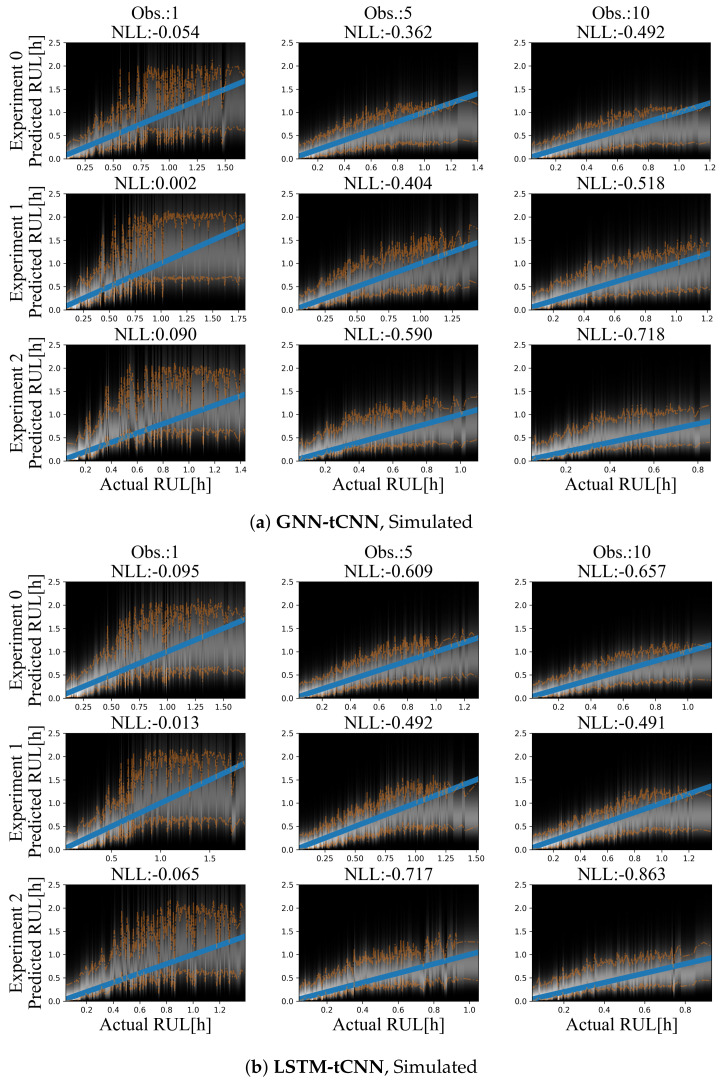
Results on the simulated dataset using (**a**) GNN-tCNN (top) and (**b**) LSTM-tCNN (bottom). The blue line corresponds to the correct prediction assuming constant linear degradation. The orange lines correspond to the 10% and 90% quantiles of the predictions.

Although it is easy to create a large number of training and test set experiments from the simulated dataset, in order to keep the simulated use-case realistic and equivalent to the bearing experiment, only 12 experiments were used for training and a set of three experiments was used as the test set. Representative prediction results for the test-set experiments are shown in Figure 9a for the GNN-tCNN and Figure 9b for the Long Short-Term Memory network with CNN feature extractor (LSTM-tCNN). The accuracy of the model is inspected in terms of the expected negative log likelihood (smaller is better). When more observations are used, the estimates for the RUL of the fictitious processes are more accurate for a larger portion of the observations, for both models. When a single observation is used, which completely neglects the long-term evolution of damage, but uses short-term features that are extracted by the learned graph-independent $\phi^{u_{0}}$ (which corresponds to a temporal CNN), the RUL estimates for both methods are inaccurate and fluctuate at the beginning of the experiment (top-left side of Figure 9a,b). Moreover, the estimated probability distributions of the RUL are narrower when approaching failure. This observation aligns with the intuition that it is not possible to have sharp estimates at initiation of the experiments.

Despite the sparsity of the observations, the degradation trend seems to be implicitly captured, when a sufficient number of observations is available. For the GNN-tCNN architecture, it may be speculated that the architecture allows for accurately capturing both the features of the high-frequency time series through the CNNs of the first graph-independent processing step, and the long-term evolution of the time series through the GraphNet processing steps.

For the simulated dataset, both methods perform on par. This supports that the GNN-tCNN approach can yield results that are competitive with the more classical RNN-based approach. The differences between the RNN-based and GNN-based approaches are mainly computational, with the GNN-model sacrificing memory efficiency due to the model’s quadradic memory cost on nodes, for circumventing the sequential computational bottleneck of RNN-based models. In this sense, the proposed GNN-tCNN scheme is more easily parallelizeable provided sufficient memory availability.

### 4.3. Bearings Dataset

**Figure 10:**
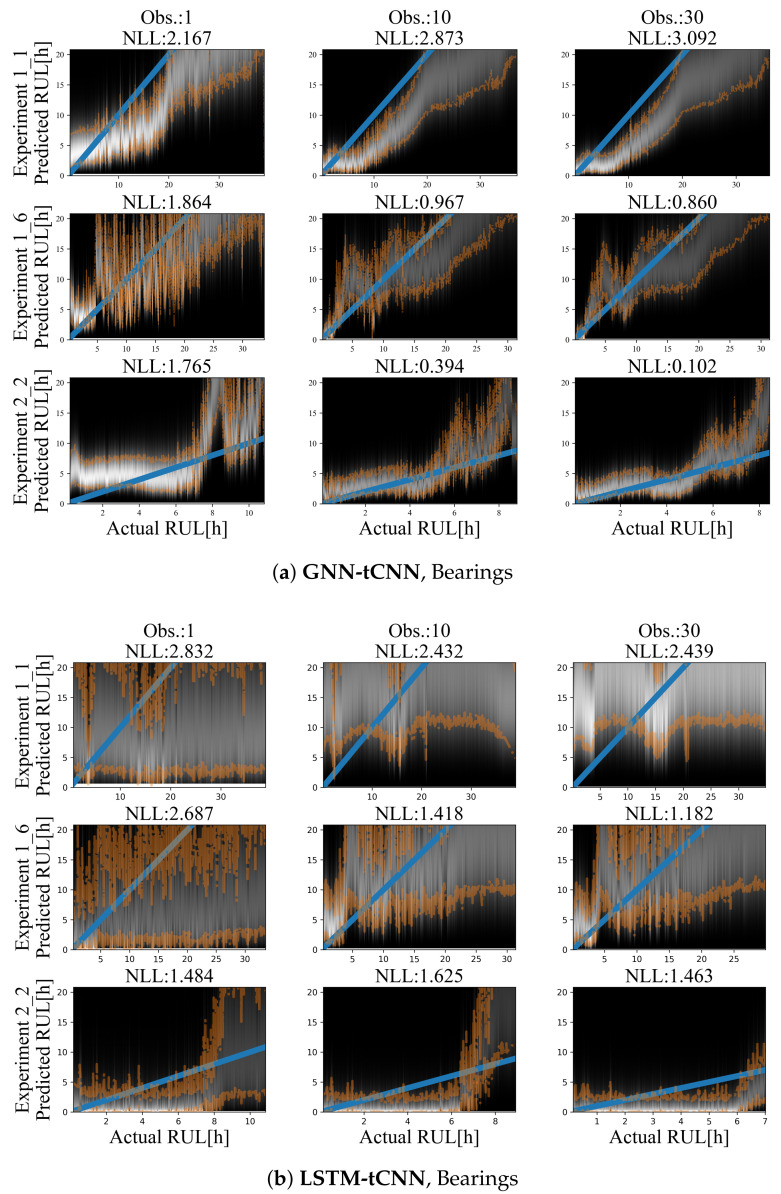
Results on the bearing dataset using (**a**) LSTM-tCNN (top) and (**b**) LSTM-tCNN (bottom). The blue line corresponds to the correct RUL assuming constant linear degradation. The orange lines correspond to the 10% and 90% quantiles of the predictions.

Results for representative experiments from the test-set of the PRONOSTIA bearing dataset are shown in Figure 10a,b and Table 5. Similarly to the simulated experiments, for both methods, predictions are characterized by smaller uncertainty closer to failure and a mostly monotonic, yet a non-linear degradation trend towards failure is predicted. The degradation trend is significantly different for each experiment. Predictions employing up to 30 arbitrarily spaced observations from the past 2000 s are shown. In both cases, the use of more observations (more than 30) did not significantly improve the accuracy or the uncertainty bounds of the predictions. This may be due to the fact that the damage phenomenon is slowly evolving, thus using a larger number of points does not offer more information on the evolution of the phenomenon.

The GNN-tCNN predictions are better overall in MAPE, but worse in NLL. The performance in the two metrics is very different and this may be attributed to the fact that MAPE divides the absolute error for a prediction with the actual RUL, hence weighing more correct predictions closer to failure, whereas the NLL metric does not take into account the magnitude of errors relative to the actual RUL. The predictions from the simple MLP model are highly inaccurate, as observed from Table 5. This inaccuracy is due to the possible inefficiency of the hand-engineered features to accurately capture damage, combined with disregarding long-term effects since the simple MLP prediction uses only a single segment.

However, if we factor in the length of the experiments and take the weighted average of those metrics, the GNN-tCNN clearly outperforms the LSTM-tCNN for this case of implementation on actual experimental data. MAPE results are reported in Table 5 for each experiment of the dataset separately, and on average, over a large number of points. The two main approaches (GNN and LSTM-based) seem to perform well in different experiments. The feature extraction and simple dropout regularized MLP-based approach did not yield good performance in all metrics.

The average MAPE over a fixed number of sub-segments of the whole time series was chosen as a stricter and more stable evaluation metric than the error in RUL estimation for a single specific point in the time series as proposed in [31]. This is the reason the reported values are larger than the ones reported in other works. Moreover, the MAPE estimate depends on deviations of the mode of the prediction and cannot take into account possible model uncertainty in a prediction, which is evidently large (as it should be) for observations that are not close to failure. The NLL performance takes uncertainty into account and therefore it is argued that it is a more appropriate evaluation metric of model accuracy. Finally, it is noted that the models with low NLL do not necessarily have low MAPE error, since MAPE is symmetric around the mode of the prediction whereas NLL takes into account the skewness of the Gamma distribution.

### 4.4. Interpretability: t-SNE Feature Visualizations

**Figure 11:**
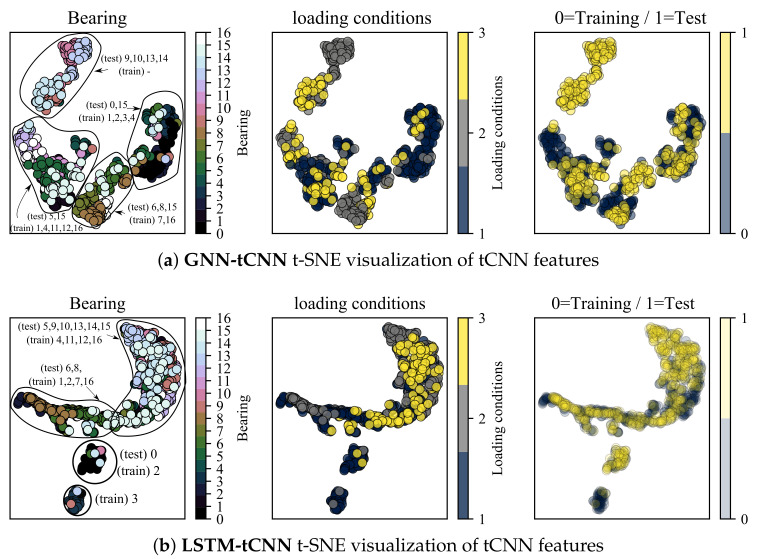
t-SNE visualization of tCNN features for segments close to failure.

In this section, the t-distributed Stochastic Neighborhood Embedding (t-SNE) dimensionality reduction technique [54] is employed to inspect the properties of feature representations of both the trained GNN-tCNN and LSTM-tCNN networks on the bearings dataset. The intermediate layer used for inspection is the output of the global pooling of the convolutional input block. The analysis is focused on features close to failure. Ideally, we want the feature representations for similar conditions of loading and degradation to lie close to each other. The t-SNE technique provides a low-dimensional representation (typically of dimension 2 or 3) of a set of higher-dimensional vectors, given some measure of similarity between these vectors. As shown in Figure 11a, discernible clusters of data are formed. These clusters correspond to experiments that produce similar sets of features for the subsequent processing with the causal GNN or LSTM. As shown in the left-most plot, embeddings from the same experiment form clusters, which is something to be expected. However, it is also observed that a number of experiments form together super-clusters, which hints to similar signals and therefore similar degradation modes for these experiments. Interestingly, the different loading conditions do not form these super-clusters in the t-SNE space, which hints to commonalities due to other underlying factors. It is possible that discrete clusters correspond to different failure modes, yet this cannot be confirmed since the failure modes of the bearings are not reported for this dataset. Interestingly the t-SNE representations of features that correspond to readings from the test-set overlap with the corresponding representations from the training set as shown in the right-most plot. This result supports the hypothesis that the tCNN features learned during the GNN-tCNN training indeed generalize well to the test-set. Furthermore, note that the clusters containing points from the experiments 2_3,2_4,2_7 and 3_1 (9,10,13,14) for the GNN-tCNN embedding does not have training data but only testing data (top left). When we do not have corresponding representations in the testing set, as it is the case for this cluster, the network is not expected to perform well. Indeed the performance for these experiments is bad for the GNN-tCNN network. Indeed, as reported in Table 5, these are 4 out of the 5 experiments in the test-set that the GNN-tCNN underperforms.

In the feature visualization of the LSTM-tCNN, the grouping of embeddings appears to be different. Firstly, two of the training experiments 1_1 and 1_4 (correspondingly 0 and 3) seem to create separate clusters from the others and this may be a sign of over-fitting. The t-SNE of 1_1 (0) overlaps with 1_3 (2) as it is the case in the GNN-tCNN embeddings. It is worth noting that the winning entry of PHM 2012 challenge [31] specifically mentions the similarity of degradation trends between experiments 1_1 and 1_3 for their hand-engineered features. In the fully data-driven models proposed herein, this similarity is learned implicitly and directly from the data, which is an approach that would scale better if more experiments and volumes of data were to be considered. The embeddings of test set experiments 6 and 8 (correspondingly 1_7 and 2_2) and training 7 and 16 (correspondingly 2_1 and 3_3) are also close for the GNN-tCNN and LSTM-tCNN features. Therefore the features used for prediction may be similar. Further evidence towards this is the similar trend predicted between the two models for the 2_2 experiment (see Figure 10), where, however, the LSTM-tCNN under-performs in the MAPE metric. The rest of the distances between the t-SNE embeddings do not agree between the two methods. The GNN-tCNN embeddings and the similarity these imply for the different models are considered more accurate, since on average in the MAPE metric the GNN-tCNN performs better.

## 5. Discussion

**Figure 12:**
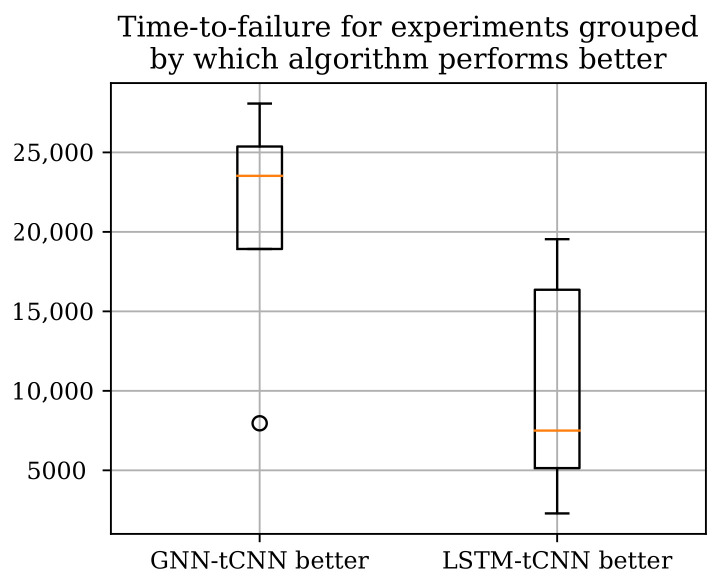
The total experiment times grouped by which RUL method performs better according to the MAE metric. The GNN-tCNN performs better for longer experiments.

Two neural network architectures, the proposed GNN-tCNN scheme and a viable alternative option RNN-tCNN (LSTM-tCNN), were applied to the problem of remaining life assessment for degradation processes from both synthetically generated, as well as actual experimental data (PRONOSTIA dataset). The two methods seem to perform well in disjoint subsets of the experiments as shown in Table 5 with the LSTM-based model performing better for the experiments that have shorter duration. The advantage of the GNN-based architecture compared against the LSTM-based architecture is that it is more efficiently exploiting distant measurements, whereas the LSTM-based method more effectively employs measurements closer to the last observation. It is thus suggested that the mechanisms of failure for the experiments where the GNN-based model performs well are gradual, whereas the cases where it underperforms are more abrupt. Assuming that gradual deterioration correlates with longer experiment times, we would expect the GNN-tCNN to perform better in longer experiments. Indeed, as shown in Figure 12 the GNN-tCNN method seems to outperform the LSTM-tCNN in experiments which result in a higher overall bearing life. This is also supported by the visualizations of the embeddings for different experiments close to failure in Section 4.4. It is not possible to completely confirm or reject this hypothesis since the accompanying documents for the PRONOSTIA dataset do not provide further details for mechanisms of failure.

The complementarity between the experiments where each of the GNN-tCNN and LSTM-tCNN methods perform well hints to further possible improvements to predictive capability of data-driven models. Such improvements may be derived through deep neural network architecture design. Both of the presented deep neural network variants are end-to-end differentiable and hence trainable with gradient-based techniques and are trained using maximum likelihood. In order to represent prediction uncertainty, a reparametrization-based technique for estimating gradients of Gamma distributions was employed. Gamma distributed outputs and a straight-forward likelihood objective are a more natural than scalar outputs and the least-squares objective commonly used in engineering, since RUL values are positive. Moreover, the predictions naturally contain uncertainty bounds, which—as expected—become narrower as the model predicts lower RUL values. Although only RUL estimation problems were considered in this work, the non-sequential causal approach to dealing with long-term dependencies may be useful for other problems where non-regularly sampled time series arise (e.g., analysis of electronic health records).

## Figures and Tables

**Table 1:** Available experiments and loading conditions.

Conditions *i*	${\dot{\phi }}_{i}$ [rpm]	$F_{i}$ [kN]	Number of Experiments
A	1800	4.0	7
B	1650	4.2	7
C	1500	5.0	3

**Table 2:** FEMTO bearings dataset, training/test split

Set	Experiment	Conditions	Failure Time [s]	Num. Obs.
Training	1_2	A	8700	871
	1_3	A	23,740	2375
	1_4	A	14,270	1428
	1_5	A	24,620	2463
	2_1	B	9100	911
	2_5	B	23,100	2311
	2_6	B	7000	701
	3_3	C	4330	434
Testing	1_1	A	28,072	2803
	1_6	A	24,470	2448
	1_7	A	22,580	2259
	2_2	B	7960	797
	2_3	B	19,540	1955
	2_4	B	7500	751
	2_7	B	2290	230
	3_1	C	5140	515
	3_2	C	16,360	1637

**Table 3:** Details on temporal CNN which acts as the node-function $\phi^{u_{0}}$ of the graph independent $GN_{enc}$ GraphNet. $n^{k},n^{s},n^{f}$ corresponds to kernel size, stride and number of filters. For dense layers $n^{f}$ corresponds to the layer width.

Layer Type	$\left(n^{k},n^{s},n^{f}\right)$	Activation
Conv1D	(1×1,1,50)	-
Conv1D	(1×3,2,18)	-
Conv1D	(1×3,2,18)	Dropout 20% ReLU
Conv1D	(1×3,2,50)	
Average Pool	(1×2,2,1)	-
Conv1D	(1×1,1,50)	-
Conv1D	(1×3,2,18)	-
Conv1D	(1×3,2,18)	Dropout 20% ReLU
Conv1D	(1×3,2,50)	-
Avg. Pool	(1×2,2,1)	-
Conv1D	(1×1,1,50)	-
Conv1D	(1×3,2,18)	-
Conv1D	(1×3,2,18)	Dropout 20% ReLU
Conv1D	(1×3,2,50)	-
Global Avg. Pool	(1×2,2,1)	-
Feed-forward	(−,−,15)	LeakyReLU

**Table 4:** Time series features used in the simple MLP approach. The features are computed separately for each accelerometer channel.

Feature	Formula
Root Mean Square	$\sqrt{E_{t}\left[{\left(x_{t}-\mu \right)}^{2}\right]}$
Kurtosis	$E_{t}\left[{\left(\frac{x_{t}-\mu }{\sigma }\right)}^{4}\right]$
Min	$\min(x_{t})$
Max	$\max(x_{t})$
Max/Min	$\min\left(x_{t}\right)/\max x_{t}$

**Table 5:** MAPE and Negative Log Likelihood (NLL) errors for test-set experiments for the bearings dataset. For both GNN-tCNN and LSTM-tCNN 15 observations over a sequence of 2000 s (or 200 segments) were used. For the simple MLP method only a single observation was used for prediction. Best values for each metric are marked in bold. Please refer to the main text for further comments. Note that in contrast to other works, the metrics reported are average for whole experiments and not for only one instant.

#exp	MAPE			NLL	
	GNN-tCNN	LSTM-tCNN	Simple MLP	GNN-tCNN	LSTM-tCNN
1_1(0)	**43.95**	353.74	393.51	3.06	**2.67**
1_6(5)	**27.96**	75.78	1326.32	**0.74**	1.21
1_7(6)	**74.14**	1701.559	202.48	5.43	**3.72**
2_2(8)	**65.37**	1717.82	3780.37	**0.02**	1.58
2_3(9)	206.56	**57.33**	1209.45	6.56	**3.31**
2_4(10)	563.05	**75.69**	436.29	14.90	**8.37**
2_7(13)	6606.83	**96.89**	101.04	76.49	**23.48**
3_2(15)	394.71	**82.57**	1179.95	11.13	**10.89**
3_1(14)	115.18	**53.21**	5478.92	4.67	**2.24**
Weighted average	**235.11**	632.61	1501.47	5.79	**3.55**

## Data Availability

Code to reproduce the experiments in the paper will be made available in https://github.com/mylonasc/gnn-tcnn, accessed on 15 September 2021.

## Abbreviations

The following abbreviations are used in this manuscript:

ML	Machine Learning
RUL	Remaining Useful Life
CNN	Convolutional Neural Network
tCNN	Temporal Convolutional Neural Network
RNN	Recurrent Neural Network
LSTM	Long Short-Term Memory
MLP	Multi-Layer Perceptron
GNN	Graph Neural Network
GNN-tCNN	Graph Network with Causal Connectivity and Temporal Convolutional Neural
	Network feature extractor
LSTM-tCNN	Long Short-Term Memory Network with Causal Connectivity and Temporal
	Convolutional Neural Network feature extractor
GN	Graph Network
t-SNE	t-distributed Stochastic Neighborhood Embedding
MAPE	Mean absolute prediction error
NLL	Negative Log Likelihood
